# Selective effects of 5-HT2C receptor modulation on performance of a novel valence-probe visual discrimination task and probabilistic reversal learning in mice

**DOI:** 10.1007/s00213-018-4907-7

**Published:** 2018-04-22

**Authors:** Benjamin U. Phillips, Sigma Dewan, Simon R. O. Nilsson, Trevor W. Robbins, Christopher J. Heath, Lisa M. Saksida, Timothy J. Bussey, Johan Alsiö

**Affiliations:** 1 Department of Psychology and Behavioural and Clinical Neuroscience Institute, University of Cambridge, Downing Street, Cambridge, CB2 3EB UK; 2 Rodent Behavioral Core, Department of Neuroscience and Physiology, Neuroscience Institute, New York University Medical Center, New York, NY 10016 USA; 3 School of Life, Health and Chemical Sciences, The Open University, Walton Hall, Milton Keynes, MK7 6AA UK; 4 Molecular Medicine Research Group, Robarts Research Institute and Department of Physiology and Pharmacology, Schulich School of Medicine and Dentistry, Western University, London, ON Canada; 5 The Brain and Mind Institute, Western University, London, ON Canada

**Keywords:** 5-HT2C receptor, SB 242084, WAY 163909

## Abstract

**Rationale:**

Dysregulation of the serotonin (5-HT) system is a pathophysiological component in major depressive disorder (MDD), a condition closely associated with abnormal emotional responsivity to positive and negative feedback. However, the precise mechanism through which 5-HT tone biases feedback responsivity remains unclear. 5-HT2C receptors (5-HT2CRs) are closely linked with aspects of depressive symptomatology, including abnormalities in reinforcement processes and response to stress. Thus, we aimed to determine the impact of 5-HT2CR function on response to feedback in biased reinforcement learning.

**Methods:**

We used two touchscreen assays designed to assess the impact of positive and negative feedback on probabilistic reinforcement in mice, including a novel valence-probe visual discrimination (VPVD) and a probabilistic reversal learning procedure (PRL). Systemic administration of a 5-HT2CR agonist and antagonist resulted in selective changes in the balance of feedback sensitivity bias on these tasks.

**Results:**

Specifically, on VPVD, SB 242084, the 5-HT2CR antagonist, impaired acquisition of a discrimination dependent on appropriate integration of positive and negative feedback. On PRL, SB 242084 at 1 mg/kg resulted in changes in behaviour consistent with reduced sensitivity to positive feedback. In contrast, WAY 163909, the 5-HT2CR agonist, resulted in changes associated with increased sensitivity to positive feedback and decreased sensitivity to negative feedback.

**Conclusions:**

These results suggest that 5-HT2CRs tightly regulate feedback sensitivity bias in mice with consequent effects on learning and cognitive flexibility and specify a framework for the influence of 5-HT2CRs on sensitivity to reinforcement.

**Electronic supplementary material:**

The online version of this article (10.1007/s00213-018-4907-7) contains supplementary material, which is available to authorized users.

## Introduction

Adaptive responding requires organisms to detect and integrate the consequences of their actions to guide future behaviour. Failure in these processes underlies decision-making impairments in numerous psychopathological conditions, including major depressive disorder (MDD) and Parkinson’s disease (Elliott et al. 1997; Frank et al. 2004). Abnormally exaggerated affective and behavioural responsivity to negative feedback is a cardinal feature of MDD, a debilitating condition characterised by multiple symptoms including persistent low mood, apathy and suicidal ideation (DSM5). Moreover, abnormalities in feedback sensitivity appear to causally contribute to the development and maintenance of MDD (Clark et al. 2009; Roiser et al. 2012), with evidence that successful antidepressant treatment may reverse this response profile (Harmer et al. 2006, 2009). This domain therefore represents a promising candidate for the development of targeted therapeutics directed at reversing cognitive profiles implicated in depressive states.

Much of the research directed at elucidating the pathophysiological basis of MDD has focussed on the serotonin (5-hydroxytryptamine; 5-HT) system (Stern 1970; Young et al. 1985). For example, previous studies have demonstrated that the short allele of the gene encoding the 5-HT transporter, SERT, mediates the development of depression following exposure to stressful life events (Caspi et al. 2003). Additionally, causal evidence indicates that depletion of tryptophan, an essential 5-HT precursor, is sufficient to evoke depressive-like symptoms in otherwise healthy humans and mice (Young et al. 1985; Franklin et al. 2012). 5-HT has also been implicated in the performance of tasks requiring feedback integration for goal-directed behaviour (Evers et al. 2005; Bari et al. 2010; Stolyarova et al. 2014), including assays of cognitive flexibility (Clarke et al. 2004; Brigman et al. 2010; Barlow et al. 2015). Thus, feedback-dependent reinforcement learning represents a strong framework for investigation of the link between emotional and cognitive dysfunction in MDD.

Despite evidence linking 5-HT with reactivity to positive and negative feedback (Rygula et al. 2015a), the mechanisms governing the influence of this neurotransmitter on this domain are not fully established. Serotonin 2C receptors (5-HT2CRs) are associated with multiple forms of feedback-dependent behaviour, including reversal learning, motivation and food intake (Boulougouris et al. 2008; Alsiö et al. 2015; Bailey et al. 2016; Xu et al. 2017; Valencia-Torres et al. 2017). Abnormalities in 5-HT2CR expression, activity and adenosine-to-inosine RNA editing have been linked with both depressive symptomology and the mechanisms of common antidepressant pharmacological interventions (Graeff et al. 1996; Pälvimäki et al. 1996; Martin et al. 2014). 5-HT2CRs, via GABAergic feedback mechanisms, are also implicated in anxiety-like behaviours (Spoida et al. 2014). As altered 5-HT2C-activity may be involved in abnormal feedback reactivity and associated symptoms, drugs with affinity for 5-HT2CRs represent therapeutic candidates for treating symptoms associated with MDD (Opal et al. 2014; Di Giovanni and De Deurwaerdère 2016).

In this study, we aimed to determine the impact of 5-HT2CRs on reinforcement feedback sensitivity by using touchscreen tasks for the assessment of probabilistic reinforcement in C57BL/6 mice. Here, we describe the development of a modified visual discrimination procedure designed to assess the impact of positive and negative feedback sensitivity on learning. This is achieved by leveraging a probabilistically reinforced ‘neutral’ stimulus presented in conjunction with standard deterministically reinforced stimuli. The pairing of a ‘neutral stimulus’ with a stimulus associated with either reinforcement or non-reinforcement allows for assessment of the contributions of positive and negative feedback to deterministic discrimination learning. We also describe the adaptation of a probabilistic reversal learning procedure previously performed in rats (Bari et al. 2010), monkeys (Rygula et al. 2015a) and humans (Murphy et al. 2003) into the mouse operant conditioning touchscreen chamber. Similar procedures have been used to assess the effects of multiple pharmacological manipulations and genetic modifications in mice (Ineichen et al. 2012; Rygula et al. 2014; Amodeo et al. 2014; Rygula et al. 2015b) and have been extensively used in human studies (Evers et al. 2005; Reddy et al. 2016). Our findings indicate that 5-HT2CRs regulate responsivity to positive and negative feedback in these tasks. These results provide a substrate for the influence of 5-HT in abnormal reactivity to feedback and further elucidate the results of previous studies that have investigated the role of 5-HT2CRs in cognitive flexibility (Boulougouris et al. 2008).

## Methods and materials

### Animals

Male C57BL/6 mice (*n* = 32) from Charles River Laboratories (Margate, UK) were housed in groups of four and used for all experiments. Animal husbandry is described in detail elsewhere (Heath et al. 2015). Animals were maintained under a 12-h reverse light cycle (light off 07:00, lights on 19:00) and were habituated to the facility for 7 days prior to the beginning of any procedures. Following habituation, all animals were weighed for 3 consecutive days to determine free-fed weights. Animals were then restricted to approximately 85% of this free-fed weight by daily provision of standard laboratory chow (RM3, Special Diet Services). Water was available in the homecage ad libitum throughout. This research has been regulated under the Animals (Scientific Procedures) Act 1986 Amendment Regulations 2012 following ethical review by the University of Cambridge Animal Welfare and Ethical Review Body (AWERB).

### Apparatus

Sixteen Bussey-Saksida mouse operant conditioning touchscreen chambers (Campden Instruments Ltd) were used for all described experiments. This apparatus has been described in full detail elsewhere (Horner et al. 2013; Mar et al. 2013; Oomen et al. 2013). A trapezoidal arena is contained within a sound-attenuating fibreboard chamber. The long wall of the chamber is composed of a touchscreen. At the other end of the chamber, the walls narrow toward a magazine with a small aperture to which the liquid reinforcer is delivered. The touchscreen is protected by a Perspex mask which contains a defined number of response apertures. The probabilistic reversal learning procedure (PRL) experiments used a three-hole Perspex mask and the valence-probe visual discrimination (VPVD) procedure used a two-hole mask. The behavioural programs were controlled by ABET II Touch software (Campden Instruments Ltd) and Whisker Server (Cardinal and Aitken 2010).

### Drugs

SB 242084 (Tocris Bioscience, Bristol, UK) and WAY 163909 (Pfizer) were selected on the basis of their selectivity profiles and previous usage in the laboratory. SB 242084 exhibits 100-fold selectivity for the 5-HT2CR over the 5-HT2BR and 158-fold selectivity for the 5-HT2CR over the 5-HT2AR (Kennett et al. 1997). WAY 163909 exhibits 46- and 20-fold selectivity for the 5-HT2CR over the 5-HT2BR and 5-HT2AR respectively (Dunlop et al. 2005). SB 242084 and WAY 163909 were dissolved in 0.9% saline at the required concentrations and volumes and frozen at − 80 °C in aliquots until required. All injections were administered intraperitoneally with a 20-min delay between injection and start of behavioural testing. All injections were administered at an injection volume of 10 ml/kg.

### Behavioural procedures

**Fig. 1:**
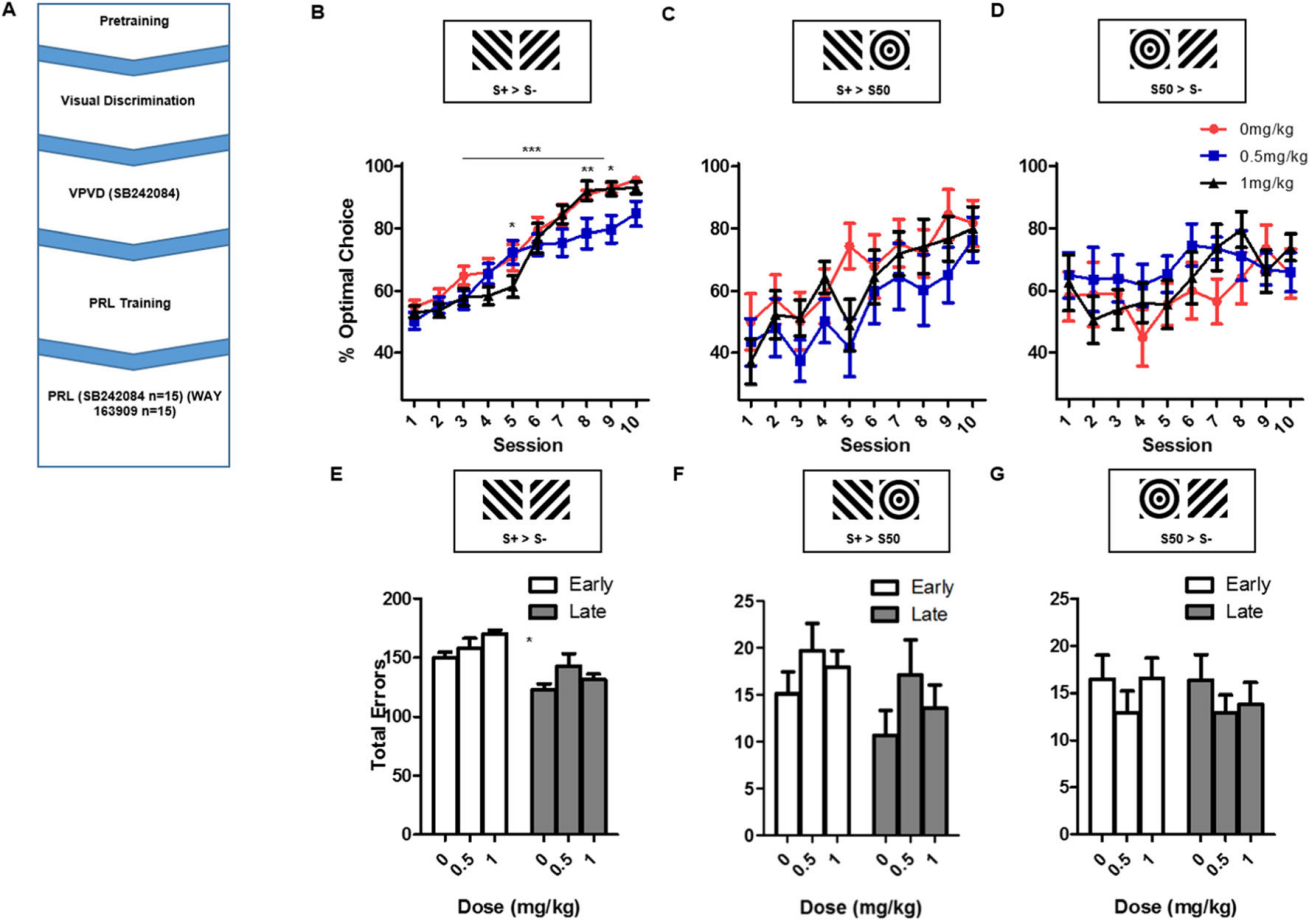
Manipulation of performance on touchscreen VPVD by administration of SB 242084. **a** Timeline of behavioural procedures and drug administration in this study. **b** Percent (mean and SEM) optimal performance on standard S+ > S− trials by session. **c** Percent (mean and SEM) optimal choice on S+ > S50 trials. **d** Percent (mean and SEM) optimal choice on S− > S50 trials. **e** Cumulative errors (mean and SEM) by phase on S+ > S− trials. **f** Cumulative (mean and SEM) errors by phase on S+ > S50 trials. **g** Cumulative (mean and SEM) errors by phase on S50 > S− trials

The order of behavioural procedures and pharmacological manipulations is displayed in Fig. 1a.

### Pre-training

Pre-training started following food restriction and was conducted as previously described (Horner et al. 2013; Mar et al. 2013; Oomen et al. 2013). All animals were habituated to the chamber environment for 20 min over 2 consecutive days. Prior to the start of these sessions, 200 μl of liquid reinforcer was manually placed in the magazine aperture. To progress to the next stage of training, all animals had to consume the reinforcer for at least one of the two sessions. Animals were then trained to associate stimuli on the screen with reinforcement for a single session. This was achieved by pairing stimulus offset with 5-μl liquid reinforcer delivery. If the animal emitted a response to the stimulus prior to offset, 15 μl of liquid reinforcer was delivered. Animals were subsequently trained to emit responses at the screen for one session. In this session, animals had to touch the stimulus to earn 5 μl of liquid reinforcer. Next, all animals were trained to initiate trials by breaking the infrared beam within the reinforcer magazine. This was conducted as per the previous stage but all animals needed to make a head entrance to the magazine for stimulus onset. Finally, animals were trained that incorrect responses resulted in a time out. In this session, any touch to the non-stimulus location resulted in a brief timeout (5 s).

### Valence-probe visual discrimination

This task is an adapted version of the previously described touchscreen visual discrimination procedure (Horner et al. 2013; Nilsson et al. 2016). Following pre-training, all animals were initially trained on a previously reported standard visual discrimination except that no correction trials were included at any stage (Horner et al. 2013). In these sessions, two visual stimuli were presented on the touchscreen concurrently. One stimulus was designated as the S+ and was reinforced 100% of the time, whilst the other was designated as the S− and never reinforced. The S+ and S− were counterbalanced between animals. For all sessions, animals were allowed to complete 60 trials. If 60 trials had not been completed after 60 min had elapsed, the session was terminated.

Once all animals had achieved 80% correct responses, probe trials were introduced. Now, the same two-choice discrimination as during the previous stage was presented on 75% of the trials. On the remaining trials, a novel stimulus was introduced and paired with either the learned S+ or S− to form two new two-choice discriminations. The novel stimulus was designated as ‘S50’. Responses to the S50 were rewarded on 50% of the trials and non-rewarded on 50% of the trials (Nilsson et al. 2015). Thus, when presented with the S+ vs S50 configuration, the S+ was the optimal response. When presented with the S− vs S50 configuration, a response to the S50 was the optimal response. All animals were required to respond optimally at 70% or more on these trials before proceeding to drug testing.

Once all animals had successfully reached criterion, a new set of discriminative stimuli (S+ and S−) were presented. The S50 stimulus remained the same. On this new discrimination, all animals were treated with SB 242084 (0, 0.5 or 1.0 mg/kg) in a between-subject design. Ten sessions of this new discrimination were presented. All animals were treated with their allocated dose 20 min before each session.

### Within-session serial probabilistic reversal learning

Following the conclusion of VPVD testing, all animals were trained on a serial probabilistic reversal learning procedure (PRL). The procedure was designed according to previously reported PRL procedure for the rat and mouse using nose-poke apparatus (Bari et al. 2010). Initially, all animals were trained a deterministic reversal learning procedure. The PRL experiments used a three-hole Perspex mask. At the beginning of each session, the two flanker locations were illuminated with white square stimuli. For every session, one of these was randomly designated as correct and one as incorrect. A touch to the correct location always resulted in the delivery of 5 μl of liquid reinforcer. Any touch to the incorrect location resulted in the omission of reinforcement and had no other programmed consequence. Between trials, an inter-trial interval of 10 s occurred. If five consecutive correct responses were emitted, the correct and incorrect locations switched, so the previously correct location became incorrect, and the previously incorrect location became correct. Animals were permitted to complete a maximum of 90 trials in a maximum of 60 min.

When performance was stable, probabilistic feedback was introduced. These sessions were identical to the deterministic reversal learning procedure described above except that the correct location was only reinforced 80% of the time. On the remaining 20% of trials, a correct response received the same feedback as an incorrect response. Conversely, incorrect responses were reinforced 20% of the time whilst the remaining 80% were treated as incorrect. Following stabilisation of performance on PRL (three consecutive sessions of no significant change in number of reversals completed), either SB 242084 (0, 0.5 or 1.0 mg/kg) or WAY 163909 (0, 1 or 3.0 mg/kg) was administered in a within-subject design, with every animal receiving vehicle and two doses of the allocated drug in a counterbalanced Latin square design on consecutive daily sessions.

### Data analysis and statistics

All data were automatically committed to a database within the ABET II Touch software. For VPVD, percent correct for each trial type was analysed. Total errors on each trial type for sessions 1–5 or 6–10 were also summed and analysed. For PRL, number of trials completed, number of reversals completed and trials per reversal were analysed. In addition, trial-by-trial analysis was used to determine win-stay and lose-shift performance by analysing the choice of animals on the trial following positive or negative feedback. Specifically, a trial was coded as win-stay if the animal chose the same location as a previously rewarded trial. A trial was coded as lose-shift if the animal switched location following a non-rewarded trial. Win-stay and lose-shift measures are expressed as conditional probabilities. In addition, response and reward collection latencies (session median per animal to reduce the influence of extreme outliers) and front and rear beam break rate (beam breaks per second) were analysed. Whole sessions were excluded from PRL analysis when the animal failed to complete the initial acquisition phase. Thus, the final *n* out of initial *n* per condition for PRL was as follows: SB 242084 0 mg/kg = 15/16, 0.5 mg/kg = 15/16, 1 mg/kg = 13/16; WAY 163909 0 mg/kg = 15/16, 1 mg/kg = 12/16, 3 mg/kg = 13/16. In addition to whole-session analysis, PRL performance was separated by task phase. Specifically, performance was calculated in isolation for ‘retention’ (all trials prior to the first five consecutive optimal responses) and ‘first reversal’ (all trials following the first five consecutive optimal responses prior to the second five consecutive optimal responses). Win-stay and lose-shift proportions for all animals were analysed using three-factor models with drug (SB 242084 or WAY 163909) as a between-subject factor and measure (win-stay, lose-shift) and dose (vehicle, low or high) as within-subject factors. Significant three-way interactions were investigated using separate two-factor models for each drug with measure and dose as within-subject factors.

Data were analysed using linear mixed models with the package ‘lme4’ in R version 3.2.2 (www.r-project.org). The Sattherthwaite approximated degrees of freedom were determined using the R package ‘lmerTest’. Significant interactions were interrogated with the Tukey adjustment for multiple comparison using the ‘lsmeans’ function from the R package ‘lmerTest’. A significance level of 0.05 was used throughout. Asterisks denote significance in all figures (*< 0.05, **< 0.01, ***< 0.005). All data are presented as mean ± standard error of the mean.

## Results

### SB 242084 impairs VPVD acquisition

Analysis of optimal choice performance on deterministic S+ > S− trials (Fig. 1b) revealed a significant main effect of session (*F*(9,234) = 77.83, *p <* 0.0001) and interaction between dose and session (*F*(18,234) = 2.32, *p* < 0.005). Post hoc analysis revealed that 0.5 mg/kg of SB 242084 impaired performance relative to vehicle at session 8 (*p* < 0.05) and that 1.0 mg/kg of SB 242084 impaired performance relative to 0.5 mg/kg at session 5 (*p* < 0.05) and facilitated performance relative to 0.5 mg/kg at session 8 (*p* < 0.01). 0.5 mg/kg of SB 242084 also impaired performance relative to vehicle and 1-mg/kg doses in session 9 (*p* < 0.05). This suggests that 0.5 mg/kg SB 242084 impaired learning in the late sessions. There was no effect of dose on S+ > S− choice trials (*F*(2,26) = 2.644, *p* = 0.09).

Analysis of performance on probe trial types together (Fig. 1c, d) revealed a main effect of session (*F*(9,520.01) = 7.17, *p* < 0.0001) and a significant dose-by-trial type interaction (*F*(2520.01) = 8.10, *p* < 0.0001) on the percentage of optimal performance. Post hoc testing revealed significantly lower performance on S+ > S50 trials in 0.5-mg/kg animals compared to 0-mg/kg treated animals (*p <* 0.005). There was also a non-significant trend for a trial type by session interaction (*F*(9,520.01) = 1.86, *p* = 0.056).

Since the significant dose-by-session interaction on S+ > S− trials appeared to be driven by different doses of SB 242084 exerting divergent effects at different stages of acquisition, we split performance into early and late sessions (< 5 sessions, > 5 sessions) and calculated the total number of errors committed during each phase (Brigman et al. 2013) (Fig. 1e–g). Analysis of these measures for non-probe S+ > S− choice trials revealed a significant effect of phase (*F*(1,26) = 56.15, *p* < 0.0001) and a significant interaction between dose and phase for incorrect responses (*F*(2,26) = 3.80, *p* < 0.05) (Fig. 1e). Post hoc pairwise comparisons did not reveal any significant dose-related effects. Analysis of S+ > S50 probe trials showed a significant effect of phase on errors (*F*(1,26) = 15.83, *p* < 0.001) (Fig. 1f) with animals making more errors in the initial sessions. No other significant effects were detected on S+ > 50 probe errors and no significant effects were detected for S50 > S− probe errors (Fig. 1g).

### SB 242084 and WAY 163909 alter sensitivity to feedback in serial PRL

**Fig. 2:**
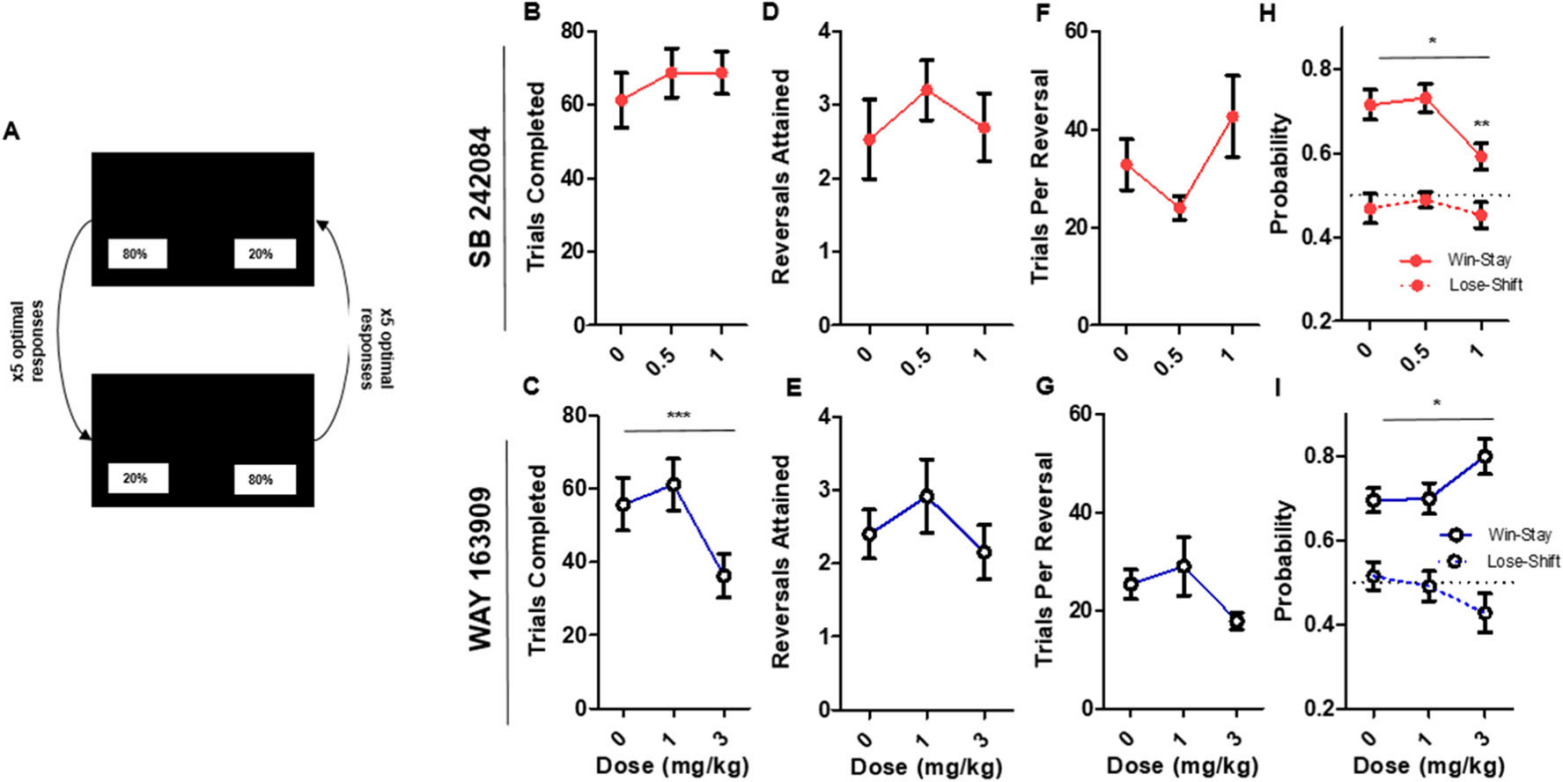
Whole-session performance on touchscreen PRL. **a** Illustration of PRL task. **b** SB 242084 overall number of trials completed (mean and SEM). **c** WAY 163909 overall number of trials completed (mean and SEM). **d** SB 242084 number of reversals attained (mean and SEM). **e** WAY 163909 number of reversals attained (mean and SEM). **f** SB 242084 trials per completed reversal (mean and SEM). **g** WAY 163909 trials per completed reversal (mean and SEM). **h** SB 242084 win-stay lose-shift conditional probabilities (mean and SEM). **i** WAY 163909 win-stay lose-shift conditional probabilities (mean and SEM)

Following VPVD, we investigated the involvement of the 5-HT2CR in reactivity to positive and negative feedback by testing animals on PRL (Fig. 2a) following acute administration of the antagonist SB 242084 and agonist WAY 163909. On the final session prior to the commencement of drug studies, an average of 2.76 ± 0.34 (SEM) reversals were completed. Animals completed an average of 64.567 ± 4.8 (SEM) trials. On feedback response measures, animals exhibited an average of 0.67 ± 0.025 (SEM) win-stay and 0.49 ± 0.02 (SEM) lose-shift.

For drug administration, on trials completed (Fig. 2b, c), there was no effect of SB 242084, but a significant main effect of WAY 163909 (*F*(2,21.77) = 9.12, *p* < 0.005) with reduced trial completion at 3 mg/kg relative to both 0 mg/kg (*p* < 0.005) and 1 mg/kg (*p* < 0.01). There was no effect of drug on overall reversals completed for either SB 242084 or WAY 163909 (Fig. 2d, e). On trials per reversal (Fig. 2f, g), there was a non-significant trend for SB 242084 in the direction of a performance impairment (*F*(2,26.58) = 2.99, *p* = 0.07).

SB 242084 and WAY 163909 significantly altered feedback response measures as measured by win-stay and lose-shift. A three-factor model containing drug (SB 242084, WAY 163909), dose (vehicle, low, high) and measure (win-stay, lose-shift) revealed a significant three-way interaction (*F*(2,154) = 5.60, *p* < 0.005) on the conditional probability measures (win-stay and lose-shift). There was also a non-significant trend toward a drug by dose interaction (*F*(2,154) = 2.44, *p* = 0.09). To investigate the three-way interaction, two-factor models (dose and measure) were fit for each drug, revealing significant effects of measure (*F*(1,80) = 67.01, *p* < 0.0001) and dose (*F*(2,80) = 4.16, *p* < 0.05) in animals administered SB 242084 (Fig. 2h). Post hoc comparison showed that 1 mg/kg of SB 242084 increased win-stay relative to vehicle (*p* < 0.01) and 0.5-mg/kg (*p* < 0.05) doses. There was also a significant effect of feedback response measure (*F*(1,74) = 70.00, *p* < 0.0001) and an interaction between this factor and dose (*F*(2,74) = 3.90, *p* < 0.05) (Fig. 2i). WAY 163909 tended to increase win-stay and reduce lose-shift choice proportions at 3 mg/kg relative to vehicle but post hoc analysis revealed no significant effects. SB 242084 and WAY 163909 administration also resulted in a set of changes in latencies and infrared beam breaks consistent with generalised changes in motoric activity (Table S1 and Fig. S1).

### SB 242084 and WAY 163909 exert opposing effects on PRL acquisition performance

**Fig. 3:**
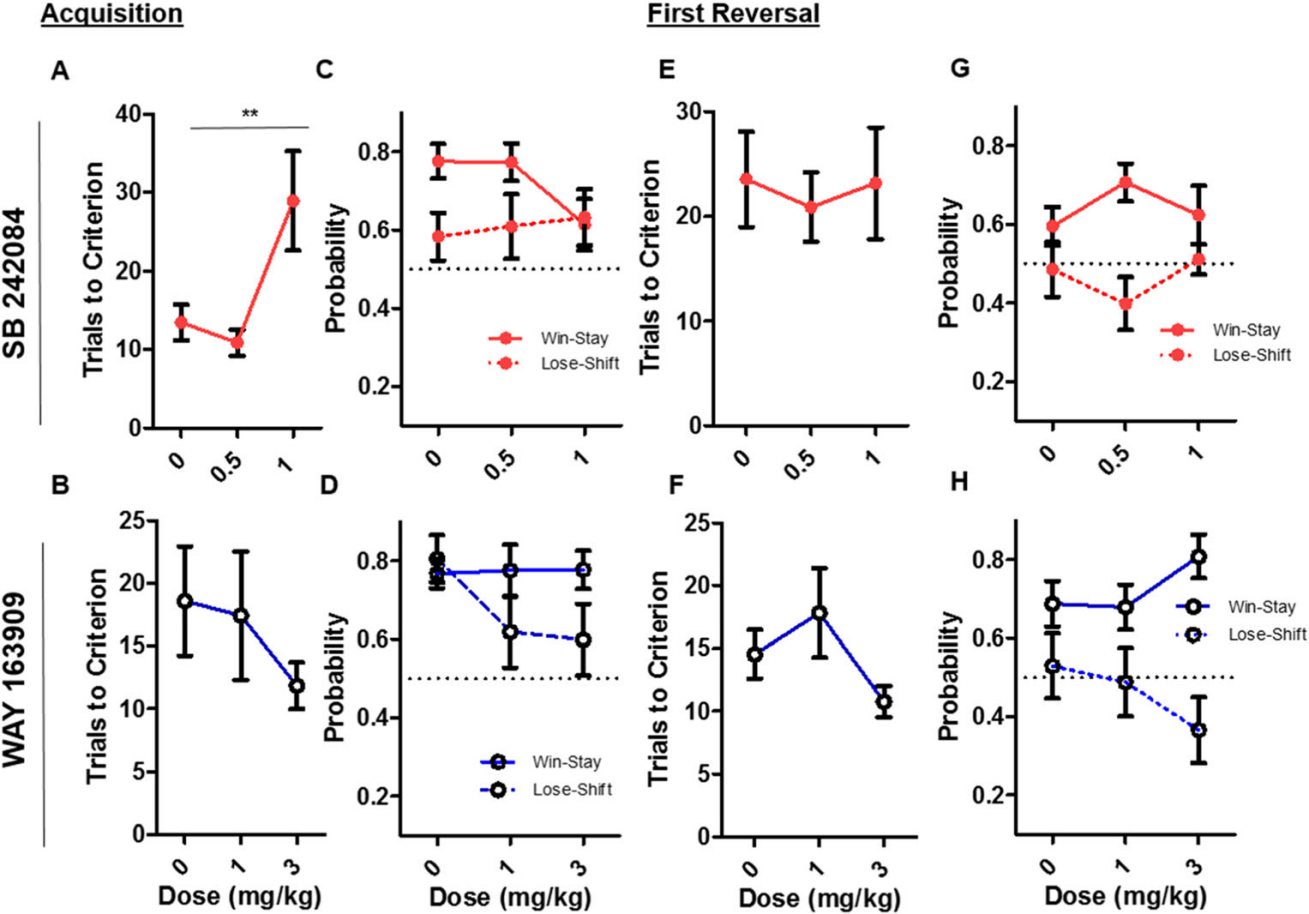
Performance on PRL separated by within-session task phase acquisition and first reversal. **a** SB 242084 acquisition trials to criterion (mean and SEM). **b** WAY 163909 acquisition trials to criterion (mean and SEM). **c** SB 242084 acquisition win-stay lose-shift conditional probabilities (mean and SEM). **d** WAY 163909 acquisition win-stay lose-shift conditional probabilities (mean and SEM). **e** SB 2424084 first reversal trials to criterion (mean and SEM). **f** WAY 163909 first reversal trials to criterion (mean and SEM). **g** SB 242084 first reversal win-stay lose-shift conditional probabilities (mean and SEM). **h** WAY 163909 first reversal win-stay lose-shift conditional probabilities (mean and SEM)

Data acquired from reversal tasks can be split into phases known to be differentially sensitive to manipulations (Bari et al. 2010; Brigman et al. 2010; Alsiö et al. 2015). To determine if 5-HT2CR manipulation resulted in task phase selective effects, we analysed acquisition and first reversal separately in the PRL procedure. This analysis revealed a significant increase in trials to criterion following SB 242084 administration (*F*(2,25.68) = 7.66, *p* < 0.005) (Fig. 3a). In contrast, no effect of WAY 163909 administration was detected on this measure (Fig. 3). For win-stay and lose-shift proportions, a three-factor model applied to the feedback response measures revealed a significant three-way interaction between drug, dose and measure (*F*(2,116.81) = 3.70, *p* < 0.05). To further investigate this effect, a two-factor model was applied. This showed a significant effect of response measure in SB 242084-treated animals (*F*(1,62.028) = 5.34, *p <* 0.05) (Fig. 3c) due to a consistently higher proportion of win-stay as compared to lose-shift. For WAY 163909-treated animals, the two-factor model revealed a non-significant trend toward a main effect of response measure (*F*(1,54.58) = 3.50, *p* = 0.067) (Fig. 3d).

Analysis of the first reversal performance revealed no significant effect of SB 242084 or WAY 163909 on trials to reversal completion (Fig. 3e, f). When the three-factor model was applied, a significant three-way interaction between drug, dose and measure was detected (*F*(2,119.04) = 3.42, *p* < 0.05). In SB 242084-treated animals, both a non-significant trend toward a significant interaction between dose and measure (*F*(1,62.52) = 2.42, *p* = 0.1)) and a significant effect of measure were detected with a two-factor model (*F*(1,62.52) = 16.06, *p* < 0.0001) (Fig. 3g). A non-significant trend toward significant interaction between WAY 163909 dose and measure was detected on feedback response measures (*F*(2,56.91) = 2.66, *p* = 0.08), as well a significant effect of response measure (*F*(1,59.31) = 62.07, *p* < 0.0001) (Fig. 3h).

## Discussion

MDD is closely associated with abnormal responsivity to negative feedback resulting in emotional and cognitive dysfunction (Elliott et al. 1997; Roiser et al. 2012). Since 5-HT is known to play a critical role in these processes (Bari et al. 2010; Ineichen et al. 2012) and discrimination learning (Izquierdo et al. 2012), we investigated the impact of systemic treatments with 5-HT2CR selective drugs on discrimination learning and positive and negative feedback responsivity in mice. Using a serial touchscreen PRL task, we show that 5-HT2CRs are involved in regulating the balance between positive and negative feedback integration in reinforcement choice procedures by affecting win-stay and lose-shift measures.

WAY 163909, a 5-HT2CR agonist, reduced the sensitivity to negative feedback and improved PRL performance. Similar to the effect of WAY 163909, high or chronic doses of SSRIs facilitate 5-HT transmission (El Mansari et al. 2005; Dankoski et al. 2016), improve reversal learning performance (Brigman et al. 2010) and decrease sensitivity to negative feedback (Bari et al. 2010) and we hypothesise that such effects may in part be mediated by altered activity at 5-HT2CRs. These results indicate that 5-HT2CRs may represent a significant locus in the mechanism of effective antidepressant therapeutics (Martin et al. 2014). Conversely, the effects of SB 2424084 on PRL appeared to be mediated by drug effects during the acquisition phase, with no detectable effects of administration of this drug on the first reversal. This is consistent with previous studies reporting an improvement in deterministic reversal learning performance following 5-HT2CR antagonist administration (Boulougouris et al. 2008; Boulougouris and Robbins 2010; Alsiö et al. 2015). Specifically, on PRL, the overall reduction in positive feedback sensitivity is hypothesised to impair acquisition performance by increasing the likelihood of switching following positive feedback. Since positive feedback is uncommon in early reversal, no change in overall reversal performance is detectable. A number of important limitations necessitate further research into the relationship between 5-HT2CR function and abnormalities in reinforcement sensitivity observed in MDD. First, these experiments were conducted in mice with an intact 5-HT system and not a putative model of MDD. The effects of the 5-HT2CR selective manipulations may differ when applied to a dysregulated 5-HT system. Therefore, future studies may seek to determine the effects of 5-HT2CR modulation on reinforcement sensitivity in candidate MDD mouse models. In addition, it is possible that the effects described in this manuscript are affected by both the order of exposure to the behavioural tasks described and the level of performance attained on PRL at the point of drug testing. However, the level of performance attained in this cohort on PRL prior to the commencement of drug administration was highly comparable to other cohorts trained in our laboratory and no effect of previous drug exposure from VPVD was detected on PRL performance. Future studies may seek to explore the interaction between training history and performance with respect to modulation of the 5-HT system.

### 5-HT2CRs as antidepressant targets

Compounds with affinity for the 5-HT2CR, typically with antagonistic mechanisms, have been shown to exert rapid antidepressant-like effects (Opal et al. 2014) and improve motivation in mice (Bailey et al. 2016) and constitute a novel antidepressant candidate. Our results, however, indicate that 5-HT2CR antagonism diminishes sensitivity to positive feedback which may potentially accentuate aspects of the negative symptomology. These results also indicate that 5-HT2CRs may play a mixed role in depressive symptomology rather than a straightforward unidirectional modulation. The precise function is likely to depend on other factors including anatomical location and RNA edited state (Dracheva et al. 2009; Lyddon et al. 2013; Valencia-Torres et al. 2017). For instance, previous studies indicate that 5-HT2CRs in the orbitofrontal cortex (Boulougouris and Robbins 2010; Alsiö et al. 2015), dorsal raphé nucleus (Spoida et al. 2014) and ventral tegmental area (Valencia-Torres et al. 2017) are likely to support highly diverse behavioural functions. Therefore, studies that seek to establish putative novel antidepressants that target 5-HT2CRs should consider carefully the mixed effect on depressive symptoms that drugs of this class are likely to evoke.

### Relevance of 5-HT2CRs and feedback sensitivity to cognitive flexibility and relationship between emotional and cognitive dysfunction in MDD

Numerous studies have established a strong link between emotional and cognitive dysfunction in MDD. Contemporary theories of MDD present a cognitive neuropsychiatric account whereby cognitive biases are closely involved in the development and maintenance of emotional dysfunction (Austin et al. 2001). Thus, cognitive abnormalities give rise to emotional dysfunction that is central to MDD via biases in reinforcement learning, environmental perception and judgement (Pinto and Whisman 1996).

Previous studies have investigated the impact of 5-HT2CRs on cognitive flexibility, specifically by using reversal learning procedures that require animals to overcome previous associations and subsequently learn a new set of contingencies. Since such procedures depend on the successful assimilation of positive and negative feedback, the results of this study may provide a powerful complement to previously reported results. Specifically, 5-HT2CR antagonism can improve reversal learning performance (Boulougouris et al. 2008; Boulougouris and Robbins 2010) by facilitating learning in the early reversal phase (Alsiö et al. 2015). It has also been reported that 5-HT2CR antagonism can impair late reversal performance (Alsiö et al. 2015). These results are consistent with the data described here. Early reversal performance is highly dependent on integration of negative feedback as the animal persists in responding to the previously correct stimulus. In late reversal performance, heightened sensitivity to positive feedback is necessary in order to make repeated similar responses and attain high levels of performance. Thus, diminished sensitivity to positive feedback via SB 242084 administration, as observed here, can impair late reversal performance by decreasing the probability a reinforced choice will be repeated. Consistently, SB 242084 impaired visual discrimination in this study. Visual discrimination does not comprise an ‘early phase’ equivalent to reversal learning resulting in SB 242084-treated animals displaying an overall impairment.

### Valence-probe visual discrimination task and touchscreen PRL

The adapted VPVD procedure, originally suggested by Nilsson et al. (2015), allows for assessment of the impact of positive and negative feedback on learning performance by leveraging a probabilistically reinforced ‘neutral’ stimulus. Mice must learn to select optimally on ‘probe’ trials where the probabilistically reinforced stimulus is presented alongside deterministically reinforced alternatives. This can be achieved by accruing independent associative strength to the stimuli, such that probe trial performance is determined by the degree of positive or negative associate strength assigned to the S+ or S− respectively. This allows positively or negatively biased reinforcement to be studied in mice in the touchscreen apparatus. Previous studies have extensively evaluated the neural and pharmacological systems governing standard discrimination learning (Winters et al. 2010; Brigman et al. 2013; Graybeal et al. 2014). However, VPVD provides insight into the processing of positive and negative feedback that determines performance on reinforcement learning procedures and may help build upon previous results that have reported discrimination phase-specific results (Brigman et al. 2010, 2013).

This report provides the first description of a touchscreen spatial probabilistic reversal learning procedure for mice. We adapted this task from previous reports (Bari et al. 2010) but optimised the reversal criterion and initially trained the mice under a deterministic reversal learning procedure. Thus, we provide methods for the assessment of probabilistic reinforcement in mice in the touchscreen apparatus and demonstrate both their sensitivity to pharmacological manipulation and the ways in which their combined use can yield insight into the relationship between reinforcement learning and biased responding to positive and negative feedback. Since rodent models have traditionally suffered from issues related to translational validity (Cryan and Holmes 2005), these tasks in combination may represent a valuable tool for the pre-clinical screening of potential therapeutics with a lower severity threshold than behavioural assays traditionally used in this area. This approach is further bolstered by recently validated touchscreen tasks for the assessment of motivation and effort related decision-making in this apparatus (Heath et al. 2015), as these domains are also critically disrupted in MDD patients and other disorders characterised by depressive symptomology (Salamone et al. 2009; Treadway et al. 2012). The operant conditioning touchscreen apparatus can therefore be used to test mice on a battery of sensitive tasks for the assessment of motivation and reinforcement learning (Markou et al. 2013).

### Conclusions

The results obtained using these novel touchscreen procedures provide a potential link between feedback response abnormalities observed in MDD with a specific function bi-directionally supported by 5-HT2CRs. Whilst 5-HT2CR antagonists increase motivation (Simpson et al. 2011) and rapidly reduce depressive-like behaviour (Opal et al. 2014), resulting in the identification of 5-HT2CRs as viable therapeutic targets for depressive symptoms (Serretti et al. 2004), the results observed in this study suggest that they may also disrupt discrimination learning and diminish sensitivity to positive feedback. These results also expand upon previous findings regarding the role of 5-HT2CRs in reversal learning by providing a positive and negative feedback integration model for performance on these tasks and provide touchscreen tasks for the assessment of these domains in mice.

## Electronic supplementary material

ESM 1 (DOCX 53 kb)
