# Supplementary material for: Selective effects of 5-HT2C receptor modulation on performance of a novel valence-probe visual discrimination task and probabilistic reversal learning in mice
Source: Psychopharmacology (Berl). 2018 Apr 22;235(7):2101–11. doi: 10.1007/s00213-018-4907-7 (PMC6015605; doi:10.1007/s00213-018-4907-7)
Supplement: Supplementary file 1 — (DOCX 53 kb) [file 213_2018_4907_MOESM1_ESM.docx]

**SUPPLEMENTARY TABLE**

Effect of SB 242084 and WAY 163909 administration on latencies and beam break rate on PRL.

| **Drug** | **Measure** | **Result** | **Post-hoc tests** |
| --- | --- | --- | --- |
| **SB 242084** | Response latency (s) (Fig.S1A) | (F(2,27.163)= 3.98, *p*<0.05) | 0mg/kg- 1m/kg  (t= 2.75, *p*<0.05) |
|  | Reward collection latency (s) (Fig.S1B) | (F(2,25.50)= 6.92, *p*<0.005) | 0mg/kg- 1mg/kg  (t= 3.69, *p*<0.05) |
|  | Front beam break rate (Fig.S1C) | (F(2,25.59)= 5.10, *p*<0.05) | 0mg/kg- 1mg/kg  (t= -2.99, *p*<0.05) |
|  | Rear beam break rate (Fig.S1C) | n.s. |  |
| **WAY 163909** | Response latency (s) (Fig.S1D) | (F(2,24.71)= 6.76, *p*<0.005) | 0mg/kg-3mg/kg  (t= -3.30, *p*<0.01)  1mg/kg-3mg/kg  (t= -3.07, *p*<0.05) |
|  | Reward collection latency (s) (Fig.S1E) | (F(2,26.76)= 5.27, *p*<0.05) | 0mg/kg-3mg/kg  (t= -2.88, *p*<0.05)  1mg/kg-3mg/kg  (t= -2.75, *p*<0.05) |
|  | Front beam break rate (Fig.S1F) | (F(2,22.42)= 7.43, *p*<0.005) | 0mg/kg-3mg/kg  (t= 3.30, *p*<0.01)  1mg/kg-3mg/kg  (t= 3.36, *p*<0.01) |
|  | Rear beam break rate (Fig.S1F) | (F(2, 21.63)= 5.24, *p*<0.05) | 0mg/kg-3mg/kg  (t= 2.82, *p*<0.05)  1mg/kg-3mg/kg  (t= 2.77, *p*<0.05) |

**SUPPLEMENTARY FIGURE LEGEND**

Latencies and beam breaks following SB 242084 and WAY 163909 administration on PRL. A) Choice response latency in seconds following SB 242084 administration (median and SEM). B) Reward collection latency in seconds following SB 242084 administration (median and SEM). C) Beam break rate (per second) for front and rear infra-red beams following SB 242084 administration (mean and SEM). D) Choice response latency in seconds following WAY 163909 administration (median and SEM). E) Reward collection latency in seconds following WAY 163909 administration (median and SEM). F) Beam break rate (per second) for front and rear infra-red beams following WAY 163909 administration (mean and SEM).

**Fig. S1**

**
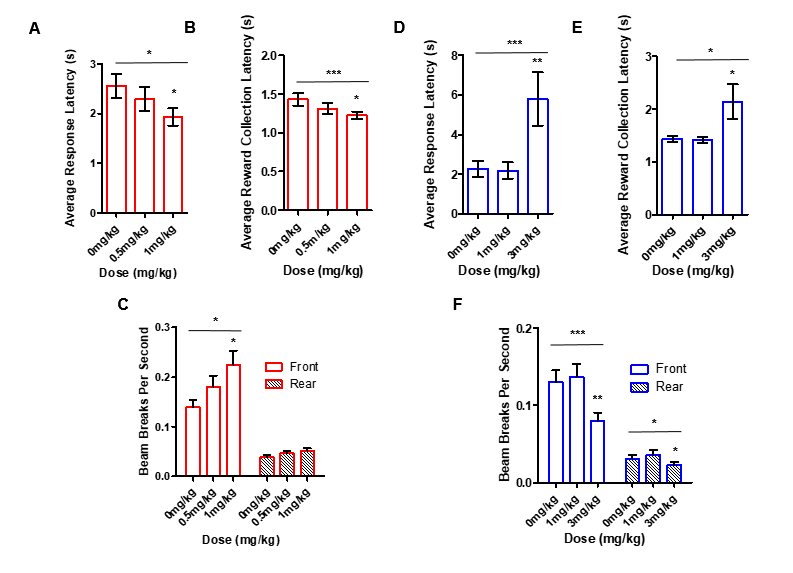
**
